# Symptom-level effects of SSRIs in depression studies in a large-scale study programme: efficacy, effects of baseline severity, impact on suicidality, and more

**DOI:** 10.1192/j.eurpsy.2023.41

**Published:** 2023-07-19

**Authors:** J. Näslund

**Affiliations:** ^1^Department of Affective Disorders, Sahlgrenska University Hospital; ^2^University of Gothenburg, Institute of Neuroscience and Physiology, Department of Pharmacology, Gothenburg, Sweden

## Abstract

**Abstract:**

Conclusions about the efficacy of antidepressants, such as the selective serotonin reuptake inhibitors (SSRIs), in clinical trials have generally been based on analyses of total sum scores on psychometric rating scales, mainly the Hamilton Depression Rating Scale (HDRS). However, this rating scale has several drawbacks, such as multidimensionality and a tendency of several items to pick up side-effects. Therefore, analyses that only rely on global drug treatment effects on the HDRS and similar scales risk misrepresenting important properties of any drugs investigated.

This presentation concerns the findings of a research project investigating item-level effects of SSRIs in treating depression in a material encompassing 8262 patients having participated in pre- and post-marketing industry-sponsored studies concering paroxetine, sertraline or citalopram. This has been a fruitful endeavour, having resulted in numerous research papers. Though our main focus at the outset was to investigate if a comparatively low efficacy of SSRIs in clinical trials was an artefact related to the use of HDRS sum scores as the main outcome (which was indeed the case), we have also used this material to investigate a number of other important questions relating to the influence of SSRIs on the various symptoms experienced by patients with depression. Major findings discussed in the presentation include: i) confirmation of early (but not widely known) reports that SSRIs significantly reduce core depression symptoms already after a week of treatment ii) strong evidence for SSRIs being equally effective in treating core depressive symptoms in mild as well as severe depression, earlier reported differences apparently being explained by differences in terms of effects (and prevalence) of non-core symptoms iii) SSRIs do not seem to exacerbate suicidality as measured by the relevant item of the HDRS scale - indeed, both average scores as well as likelihood of worsening are significantly reduced already at week 1. The implications of these, and other, findings are discussed in relation to both the role of SSRIs in the clinic as well as pre-clinical research into e.g. the mode of action of these drugs.

**Disclosure of Interest:**

None Declared

